# The Anti-Inflammatory Activity of Toonaciliatin K against Adjuvant Arthritis

**DOI:** 10.1155/2017/9436280

**Published:** 2017-10-17

**Authors:** HaiXing Gou, Jie Ye, YiRu Wang, XiaoLi Xu, QiXing Shen, JingWei Xue, Jie Zhao, XinGang Lu

**Affiliations:** ^1^Department of Traditional Chinese Medicine, Shanghai Key Laboratory of Clinical Geriatric Medicine, Huadong Hospital, Fudan University, Shanghai 200040, China; ^2^Department of Orthopaedics, Longhua Hospital, Shanghai Traditional Chinese Medicine University, Shanghai 200032, China; ^3^Department of Emergency, Jiading Hospital of Traditional Chinese Medicine, Shanghai 201899, China; ^4^Department of Traditional Chinese Medicine, Sixth People's Hospital, Jinshan Branch, Shanghai 201500, China

## Abstract

Toonaciliatin K is a natural limonoid purified from the* Toona ciliata* Roem. var. ciliata (Meliaceae). This study is to reveal the inflammatory suppression effect of toonaciliatin K and further the intrinsic mechanism. Firstly, anti-inflammatory effect of toonaciliatin K was evaluated in lipopolysaccharide- (LPS-) induced RAW264.7 cells. RT-PCR results indicated that the mRNA expressions of TNF-*α*, IL-6, and IL-1*β* were downregulated by toonaciliatin K. The toonaciliatin K inhibited TNF-*α*, IL-6, and IL-1*β* levels stimulated by LPS. Furthermore, LPS elicited the excess iNOS and COX-2 mRNA and protein production and toonaciliatin K attenuated the excess production. Western blot assay demonstrated that MAPK and NF-*κ*B signaling pathways play critical roles in the toonaciliatin K's anti-inflammatory activity. Secondly, toonaciliatin K inhibited carrageenan-induced paw edema in rats. Thirdly, toonaciliatin K alleviated the paw swelling and improved arthritis clinical scores in the adjuvant arthritis rats. Toonaciliatin K decreased the proinflammatory cytokines levels and Mankin scores in adjuvant arthritis rats. The HE staining, safranin O-fast green, and toluidine blue staining results demonstrated that toonaciliatin K alleviated the histological changes of paw, for example, pannus formation, focal loss of cartilage, bone erosion, and presence of extra-articular inflammation. Hence, toonaciliatin K is a promising agent for treatment of arthritis.

## 1. Introduction

Rheumatoid arthritis (RA) is a frequent joints disease which affects elderly people the most [1]. High morbidity and severity have led to an acute decline in quality of life worldwide. The arthritis is an inflammation-related disease [2]. The main feature of arthritis, especially RA, is synovial joint inflammation and cartilage destruction [3]. Many cytokines play crucial roles in the pathogenesis of inflammatory arthritis such as tumor necrosis factor alpha (TNF-*α*), interleukin-6 (IL-6), and interleukin-1*β* (IL-1*β*) [4]. The strategy of arthritis treatment commonly focuses on the inflammation regulation and made many contributions [5]. However, current arthritis therapy remains limited and unsatisfactory. The long-term abuse of nonsteroidal anti-inflammatory drug, which accounts for the main treatment of arthritis, resulted in numerous side effects, for example, cardiovascular disease, gastrointestinal disorder, and renal irritations [6]. Plant therapy, as one of the old and widely treatment systems, focused on the prevention and cure of disease in long history, especially in moderate disorders and chronic and recurrent infections [7]. New compound isolation, screening, and transformation with definite chemical structure from natural plants were proved as a shortcut to find effective anti-inflammatory drugs in the past years [8].

Limonoids were main part of secondary metabolites in citrus fruit and juice since the first identification as the bitter substance of citrus seeds in 1841 [9]. The kingdom of natural limonoids mainly presented in Meliaceae and Rutaceae and also frequently presented in Cneoraceae and* Harrisonia *sp. of Simaroubaceae [10]. The limonoids were used as antibacterial, antifungal, and antimalarial in juice industry in the past [11]. Nowadays, phytochemical studies have revealed that limonin and limonoids exerted pharmacology ability of anticancer [12, 13], antiobesity [14], anti-inflammatory [15], and hepatoprotective [16]. The limonoids genus has made significant progress for the chemoprevention of cancer, low back pain, and wounds [17]. Some limonoids, named toonacilianins A–I, were found in* Toona ciliata,* a plant species cultivated worldwide [18]. Two norlimonoids (toonacilianins K and L) with similar structure were isolated from the* Toona ciliata* Roem. var. ciliata (Meliaceae) by Liu and his team [19]. In Liu's report, the cytotoxicity of isolated limonoids was tested on A549 and HL-60 cells also. The 5a, 6b, and 8a-trihydroxy-28-norisotoonafolin exhibited the most sensitive cytotoxicity, while toonaciliatin F exerted weak cytotoxicity against the above two carcinoma cell lines. However, the toonaciliatin K did not exhibit apparently cytotoxicity in Liu's report. In our research, we examined the anti-inflammatory ability of toonaciliatin K on lipopolysaccharide- (LPS-) treated murine macrophages RAW264.7 cells, carrageenan-induced paw edema, and adjuvant induced-arthritis rats. The underlying mechanism of toonaciliatin K's anti-inflammatory ability was also investigated.

## 2. Material and Methods

### 2.1. Reagent, Agent, Cell Culture, and Animals

Toonaciliatin K standard preparation (purity > 98%) was provided from National Institute for the Control of Pharmaceutical and Biological Products (Beijing, China). Protein and RNA extraction kit and dimethyl sulfoxide (DMSO) were purchased from Beyotime Institute of Biotechnology (Beyotime, Haimeng, China). The 3-(4,5-dimethylthiazol-2-yl)-2, 5-diphenyl-2H-tetrazolium bromide (MTT), LPS, dexamethasone (DEX), carrageenan, indomethacin (IND), and Freund's complete adjuvant (FCA) were purchased from Sigma-Aldrich (St. Louis, USA). The enzyme-linked immune-sorbent assay (ELISA) kits were gained from R&D (R&D Systems, Minneapolis, MN). Antibodies were acquired from Cell Signaling Technology (CST, Beverly, USA).

RAW264.7, HEK293, and L02 cells were purchased from the Cell Bank of Chinese Academy of Sciences (Shanghai, China). Cells were maintained in a humidified atmosphere of 5% CO_2_ at 37°C with commercial Dulbecco's modified eagle's medium (DMEM) containing 10% fetal bovine serum (FBS) (Gibco, Carlsbad, USA). Toonaciliatin K was dissolved in DMSO with final concentration less than 1% for cell study.

Current animal study was strictly conducted following Declaration of Helsinki and the Guide for Care and Use of Laboratory and approved by the Institutional Animal Care and Use Committee of Fudan University (Grant number 2016K1036). Sprague-Dawley (SD) rats at 150–170 g with all males from Slac Animal Corporation were kept in animal facility of Shanghai Key Laboratory of Clinical Geriatric Medicine with a temperature of 25 ± 3°C, humidity of 40 ± 5%, and also a 12 h light and 12 h dark cycle environment with abundant diet and water. Toonaciliatin K was dissolved in sterilized 5% DMSO/saline for rat study. A total of 228 rats were used in this experiment. 120 rats were used in acute toxic study (60 males and 60 females). 40 rats were used in carrageenan-induced paw edema experiment (all males). 48 rats were used in acute toxic study adjuvant arthritis experiment (all males).

### 2.2. Cell Research

#### 2.2.1. MTT Assay

Cytotoxicity of toonaciliatin K on RAW264.7 cells was measured via MTT assay as described [20]. Concisely, RAW264.7 cells were cultured in 96-well plates and induced by toonaciliatin K (0 to 250 *μ*M) for 24 h. Cell viability under toonaciliatin K exposure was calculated using our previous method [20]. In addition to human tumor cells, human embryonic kidney (HEK293) cells and human hepatic (L02) cells were employed to explore toonaciliatin K's cytotoxicity under the same dose range and expose time on human normal cells [21].

#### 2.2.2. Study Design and Toonaciliatin K Concentrations

Cell study followed previous literature with minor changes [22]. Cells were divided into six groups: (1) the saline group without LPS and toonaciliatin K (control), (2) LPS group without toonaciliatin K coexposure (LPS), (3) LPS-induced group with DEX coexposure (0.5 *μ*g/mL) (positive, DEX) as previous report [22], (4) LPS group with toonaciliatin K coexposure (7 *μ*M), (5) LPS group with toonaciliatin K coexposure (14 *μ*M), and (6) LPS group with toonaciliatin K coexposure (28 *μ*M). After 12 h LPS and toonaciliatin K coexposure, cells and supernatants were collected for consequent examination.

#### 2.2.3. ELISA Assay

After LPS stimulation and toonaciliatin K incubation, cultured medium in each well was collected and centrifuged at 1000*g* for 10 min at 4°C as soon as possible. The inflammatory cytokines levels of TNF-*α*, IL-6, IL-1*β*, and the nitric oxide (NO) levels were measured with ELISA kits following the manufacture instructions separately.

#### 2.2.4. Real-Time PCR

After LPS stimulation and toonaciliatin K incubation, RNA in RAW264.7 cell was extracted using Trizol, and cDNA was conducted with a SuperScript III First-Strand Synthesis System (Invitrogen Life Technologies). For amplification, qPCR reaction solutions were composed of 50 ng of cDNA, 200 *μ*M of each primer, and SYBR® Premix Ex Taq™ (1x) in a volume of 20 *μ*L with 30 cycles of 10 s at 95°C, 10 s at 60°C, and 30 s at 72°C. The primer sequences and the probe-sequence were indicated in Table 1.

#### 2.2.5. Western Blotting

To analyze the effect of toonaciliatin K on underlying inflammatory-related signaling pathway, western blotting was used following the previous method [23]. The primary antibodies were used for determination of protein in RAW264.7 cells. The Histone-H3 was conducted for the control of nuclear protein and *β*-actin was conducted for the control of total protein.

### 2.3. Animal Research

#### 2.3.1. Acute Toxicity

The acute toxicity study was performed in rats according to the OECD guidelines (TG-420) [24]. Rats were allocated into test group and vehicle group randomly with half male and female. For test group, the toonaciliatin K was dissolved in the 5% DMOS/saline and injected into rats via tail vein. Ten rats with half male and female were used for test of each injection dose. Meanwhile, another group of 10 rats with half male and female was set as vehicle group. The same volume of 5% DMOS/saline was injected into vehicle group. These signs such as change or pause in the respiratory rhythm, convulsions, areflexia, analgesia, vomiting, uncontrolled urination, uncontrolled defecation, and death were recorded as toxicity signs [25]. The acute toxicity assay was initiated from 1 mg/kg to probe safety dose range of toonaciliatin K (i.v.) as in previous literature [26]. The dose range was set as 1, 20, 50, 100, 200, 300, 400, and 500 mg/kg. If there were no signs of toxicity or no diminished activity in total of 72 h, then this dose was recorded as safe dose. After that, the following dose was tested. If there were any signs of toxicity or no diminished activity, then this dose was recorded as the maximum tolerance dose of toonaciliatin K and further dose will not be proceeded.

#### 2.3.2. The Carrageenan-Induced Paw Edema

Furthermore, according to previous report [27], carrageenan-triggered paw edema assay was performed to explore the anti-inflammatory activity of toonaciliatin K. Rats were allocated into five groups randomly (*n* = 8): vehicle group, 3 test groups, and positive group. Test group was injected with toonaciliatin K via tail vein at three dosages (8.3, 16.5, or 33 mg/kg). DEX (5 mg/kg) was injected into positive while the same volume saline into vehicle [26]. 4 h later, carrageenan (0.1 ml; 1% w/v in saline) was injected into subplantar of right hind paw of rats in all groups. Paw edema volume was measured using plethysmometer at five desired points (0, 1, 2, 3, 4, and 5 h after injection) and recorded as volumes of (desired group/control group) *∗* 100% following previous procedure [28].

#### 2.3.3. The Adjuvant Arthritis

In this animal experiment, rats were randomized and enrolled into six groups (*n* = 8): control group vehicle group, three toonaciliatin K (8.3, 16.5, and 33 mg/kg) groups, and positive group. Naïve rats were used as control. Adjuvant arthritis model was established by FCA injection according to previous report [29] in all groups except control group. Seven days after injection of FCA, toonaciliatin K was injected via tail vein at three dosages (8.3, 16.5, or 33 mg/kg) every three days during dosing period. The course of toonaciliatin K administration lasted 27 days. Meanwhile, 5% DMSO/saline was injected via tail vein as vehicle. Animals in positive group were orally treated with IND (once daily treated at 20 mg/kg) [30]. The animals in control groups received neither FCA nor DMSO.

#### 2.3.4. Hind Paw Swelling and Arthritis Clinical Scores

Hind paw swelling rate was analyzed after the establishment of adjuvant arthritis model following the reported method [29]. The hind paw volumes were recorded using plethysmometer each 3 days from the first toonaciliatin K administration day. Increasing of paw swelling (%) was measured according to the formula: (increased multiples of right hind paw volume) − (basic hind paw volume)/(basic hind paw volume). Arthritis clinical scores were analyzed each 3 days following previous literature [31].

#### 2.3.5. ELISA Assay in Serum

Briefly, adjuvant arthritis rats were sacrificed after last toonaciliatin K administration. Rat blood was collected in blood collection tubes, centrifuged at 4000 rpm/min for 10 min at 4°C. After that, supernatant of blood was stored in −80°C until examination. Proinflammatory cytokines levels about TNF-*α*, IL-1*β*, and IL-6 in rat serum were measured using ELISA kits separately.

#### 2.3.6. Rats Paw Histological Examination and Scores

After execution, the right paws were dissected from adjuvant arthritis rats kept intact for histological examination and the soft tissues of paw were isolated carefully. Paw tissues were fixed immediately and decalcified for 1.5 month. Then the tissue specimens were dehydrated, embedded in paraffin, and cut into 5 *μ*m sections. The paw sections were stained using hematoxylin-eosin (HE) and safranin O-fast green for histological assay. The arthritis histological scores regarding HE staining were analyzed by individual double-blind pathologist: 0 (normal), 1 (mild swelling), 2 (moderate swelling), 3 (severe swelling), and 4 (excess swelling with joint rigidity) [32]. Degree of arthritis histological change regarding safranin O-fast green staining was analyzed by individual double-blind pathologist using Mankin scoring system as Zhong's literature [33]. Furthermore, paw sections were stained with toluidine blue stain solutions (1%) for the evaluation of sulfated glycosaminoglycan (CAG) synthesis as Salvatore's literature [34]. Evaluation was performed based on the staining intensity. All images were captured with microscope (D5100, Nikon, Tokyo, Japan).

### 2.4. Analysis of Data

Triplicate experiments were obtained by independent samples unless otherwise mentioned. The results were made as means ± standard deviation (SD) and determined using one-way ANOVA followed with Bonferroni test. All analyses were conducted using software with significance at <0.05 (SPSS19.0, Chicago, IL).

## 3. Results

### 3.1. Cell Results

#### 3.1.1. The Cytotoxicity

**Figure 1:**
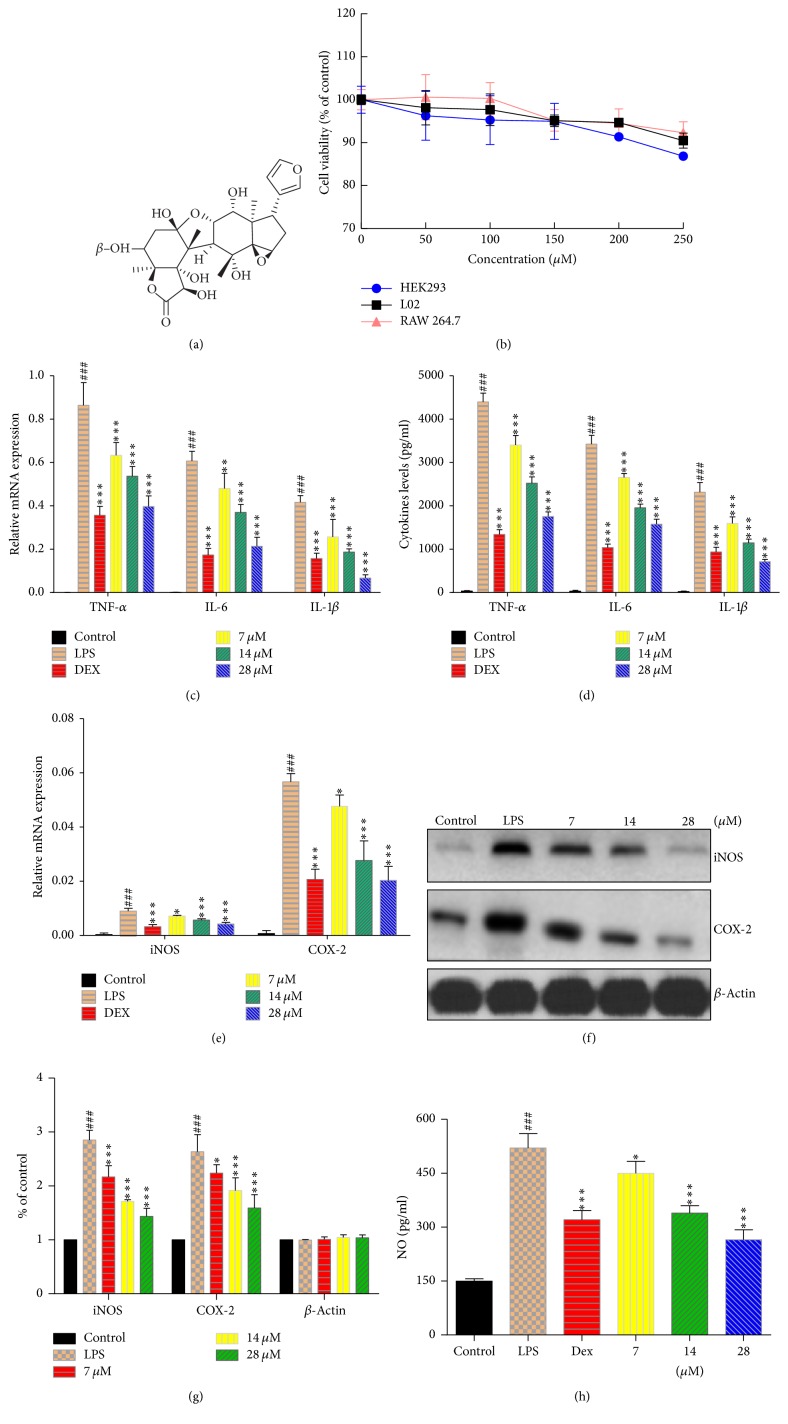
The chemical structure, safety, and anti-inflammatory activities of toonaciliatin K on RAW264.7 cells. (a) The chemical structure of toonaciliatin K. (b) The MTT assay using toonaciliatin K at 24 h exposure on RAW264.7, HEK293, and L02 cells. For consequent assay, the RAW264.7 cells were treated with toonaciliatin K exposure (7, 14, or 28 *μ*M) combined with LPS (10 ng/mL) for 12 h. The dexamethasone (Dex, 0.5 *μ*g/mL) was used as positive. There were no LPS and toonaciliatin K in control group. (c) The mRNA expression levels of TNF-*α*, IL-6, and IL-1*β*. (d) The cytokines levels of TNF-*α*, IL-6, and IL-1*β*. (e) The mRNA levels of iNOS and COX-2. (f) The representative protein levels of iNOS and COX-2. (g) The quantification of protein levels of iNOS and COX-2. (h) The NO production levels. Statistical differences in different concentrations were considered significant at the levels of ^*∗*^*P* < 0.05, ^*∗∗*^*P* < 0.01, or ^*∗∗∗*^*P* < 0.001. The statistical differences among LPS group and control group were considered significant at the levels of ^###^*P* < 0.001.

Exposure of toonaciliatin K at 0–250 *μ*M for 24 h did not exert impact on the cell viability of RAW264.7 (Figure 1(b)). What is more, toonaciliatin K exerted significantly low cytotoxicity on 2 types of human normal cells (HEK 293: kidney cell line; L02: liver cell line) at 0–250 *μ*M for 24 h exposure (Figure 1(b)). MTT results implied that toonaciliatin K is safe on human normal cells, although some similar compounds exerted weak cytotoxicity activity on carcinoma cells in previous report.

#### 3.1.2. Effect of Toonaciliatin K on Proinflammatory mRNA Expression, Proinflammatory Cytokines Levels, and NO Levels in LPS-Stimulated RAW264.7 Cells

To evaluate the effect of toonaciliatin K on the expression of proinflammatory cytokines, the levels of TNF-*α*, IL-6, and IL-1*β* were assessed by RT-PCR. The levels of TNF-*α*, IL-6, and IL-1*β* maintained low levels in unstimulated RAW264.7 cells (Figure 1(d)). LPS exposure resulted in the significant upregulation of TNF-*α*, IL-6, and IL-1*β* mRNA expression (Figure 1(c)). Furthermore, the cytokines levels about TNF-*α*, IL-6, and IL-1*β* were increased after LPS stimulation (Figure 1(d)). However, the coexposure treatment of toonaciliatin K at 7, 14, and 28 *μ*M for 12 h inhibited the increase of mRNA expression of TNF-*α*, IL-6, and IL-1*β*. The proinflammatory cytokines levels of TNF-*α*, IL-6, and IL-1*β* were also inhibited by toonaciliatin K in a concentration-dependent manner.

To further investigate the effect of toonaciliatin K on NO production, the mRNA and protein levels of inducible nitric oxide synthase (iNOS) and cyclooxygenase-2 (COX-2) were determined and quantified. Compared with normal cells, LPS exposure successfully induced the mRNA expression of iNOS and COX-2 (Figure 1(e)). As shown in Figure 1(e), toonaciliatin K attenuated the increase of the mRNA expression of iNOS and COX-2. Western blot results indicated that toonaciliatin K inhibited iNOS and COX-2 protein expression in a dose-dependent manner (Figures 1(f) and 1(g)). ELISA results demonstrated that toonaciliatin K inhibited the NO production in a concentration-dependent manner (Figure 1(h)).

#### 3.1.3. Effect of Toonaciliatin K on Inflammatory-Related Signaling Pathways

**Figure 2:**
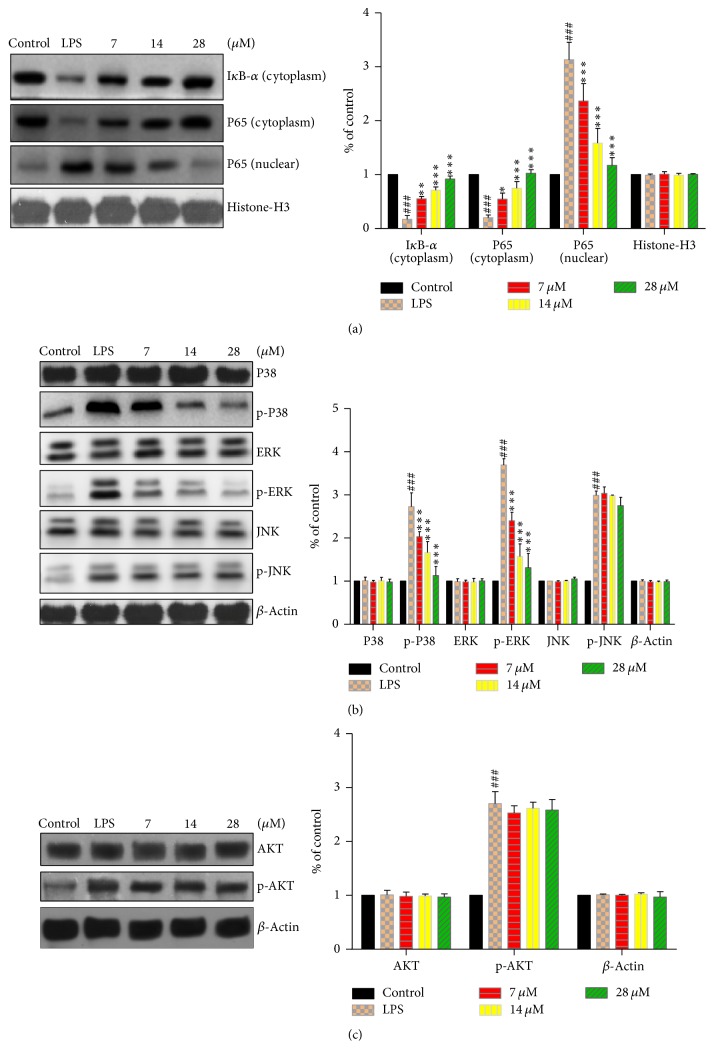
The signaling pathways involved in the anti-inflammatory activities of toonaciliatin K in vitro. The RAW264.7 cells were induced with toonaciliatin K exposure (7, 14, or 28 *μ*M) combined with LPS (10 ng/mL) for 12 h. The dexamethasone (Dex, 0.5 *μ*g/mL) was conducted as positive. The control group received no LPS and toonaciliatin K. (a) The NF-*κ*B signaling pathway activity and its quantification. (b) The MAPK signaling pathway activity and its quantification. (c) The AKT signaling pathway activity and its quantification. Statistical differences in different concentrations were considered significant at the levels of ^*∗*^*P* < 0.05, ^*∗∗*^*P* < 0.01, or ^*∗∗∗*^*P* < 0.001. The statistical differences among LPS group and control group were considered significant at the levels of ^###^*P* < 0.001.

The mitogen-activated protein kinase (MAPK) and nuclear factor-kappa B (NF-*κ*B) signaling pathway are usually inflammatory-involved pathways [35]. Meanwhile, the phosphoinositide 3-kinase (PI3K)/AKT signaling pathway often takes part in the inflammatory activity in LPS-induced RAW264.7 cells [36]. In the LPS-stimulated RAW264.7 cells, LPS triggered the activation of NF-*κ*B and phosphorylation of P38, extracellular regulated protein kinases (ERK), c-Jun N-terminal kinase (JNK), and AKT in RAW264.7 cells. The toonaciliatin K treatment leaded to the attenuation in NF-*κ*B expression levels (Figure 2(a)) and ERK and p38 phosphorylation (Figure 2(b)). However, the phosphorylation JNK and total JNK were not affected by toonaciliatin K treatment with three concentrations (Figure 2(b)). In addition, toonaciliatin K treatment with three concentrations exhibited no inhibitory effect on the LPS-induced increase of the phosphorylation level of AKT (Figure 2(c)).

### 3.2. Animal Results

#### 3.2.1. Evaluation of Toxicology and Safety

In the acute toxic test, the toxic signs such as vomiting, tachypnea, and diminished activity occurred in rats when the dose of toonaciliatin K reached 300 mg/kg (i.v.). Then 300 mg/kg was recorded as maximum tolerance dose. However, the toonaciliatin K was injected using three dosages (8.3, 16.5, and 33 mg/kg) in the carrageenan-induced paw edema and adjuvant arthritis rats. Therefore, the toonaciliatin K (i.v.) dose range used in further animal experiments, which is nearly 2–10 percent of maximum tolerance dose (tested dose of 8.3 mg/kg, 16.5 mg/kg, and 33 mg/kg versus maximum tolerance dose of 300 mg/kg), was relatively safe.

#### 3.2.2. Anti-Inflammatory Ability of Toonaciliatin K on Carrageenan-Induced Paw Edema in Rats

**Figure 3:**
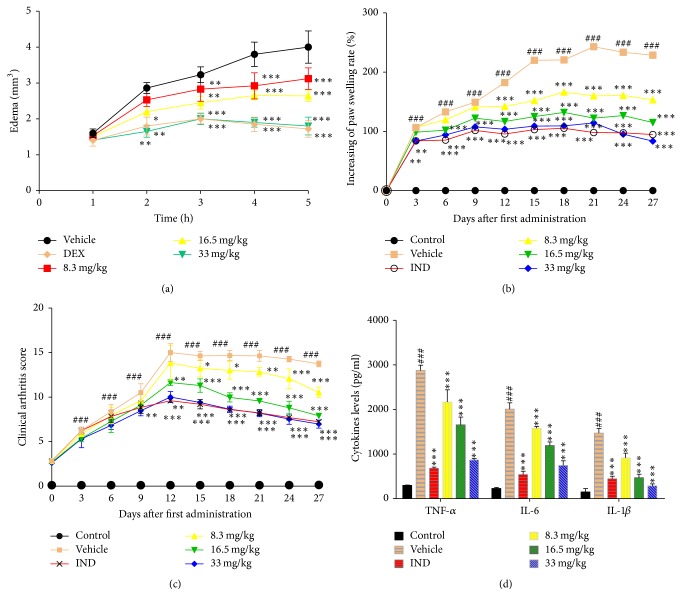
The antiedema effect of toonaciliatin K in carrageenan-induced paw edema SD rats. Rats were pretreated with toonaciliatin K (8.3, 16.5, or 33 mg/kg, iv) for 4 h. The DEX (5 mg/kg, p.o) was administrated for 24 h as positive group. The same volume of 5% DMSO/saline was injected into vehicle. (a) Effect of toonaciliatin K pretreatment on carrageenan-induced rats paw edema. Furthermore, the antiadjuvant arthritis effect of toonaciliatin K in rats was tested. Toonaciliatin K was injected using tail vein at the dosage of 8.3, 16.5, or 33 mg/kg each three days in total of 27 days of administration. The same volume 5% DMSO/saline was injected into vehicle. The IND (20 mg/kg) was administered orally once a day as positive. There was no intervention to control group. (b) The paw swelling increased in each group after toonaciliatin K administration. (c) The arthritis clinical scores in each group after toonaciliatin K treatment. (d) The expression level of proinflammatory cytokines in each group after toonaciliatin K treatment. Statistical differences in different concentrations were considered significant at the levels of ^*∗*^*P* < 0.05, ^*∗∗*^*P* < 0.01, or ^*∗∗∗*^*P* < 0.001. The statistical differences among vehicle group and control group were considered significant at the levels of ^###^*P* < 0.001.

The carrageenan induced a significant paw edema in the vehicle group. As shown in Figure 3(a) toonaciliatin K administration (i.v.) inhibited the paw edema in carrageenan-induced paw edema in a dose-dependent manner.

#### 3.2.3. Inhibition Ability of Toonaciliatin K on Adjuvant Arthritis Rats

The FCA elicited marked paw swelling and resulted in significant increase in the arthritis clinical scores of rats in the vehicle group. However, toonaciliatin K alleviated the paw swelling (Figure 3(b)) and possessed a downregulation trend in the arthritis clinical scores in adjuvant arthritis rats (Figure 3(c)) in a dose-dependent manner. The proinflammatory cytokines of TNF-*α*, IL-6, and IL-1*β* in serum of adjuvant arthritis rats increased in the vehicle group. Toonaciliatin K administration attenuated the proinflammatory cytokines in a dose-dependent manner (Figure 3(d)).

#### 3.2.4. Effect of Toonaciliatin K on Histological Changes of Adjuvant Arthritis Rats

**Figure 4:**
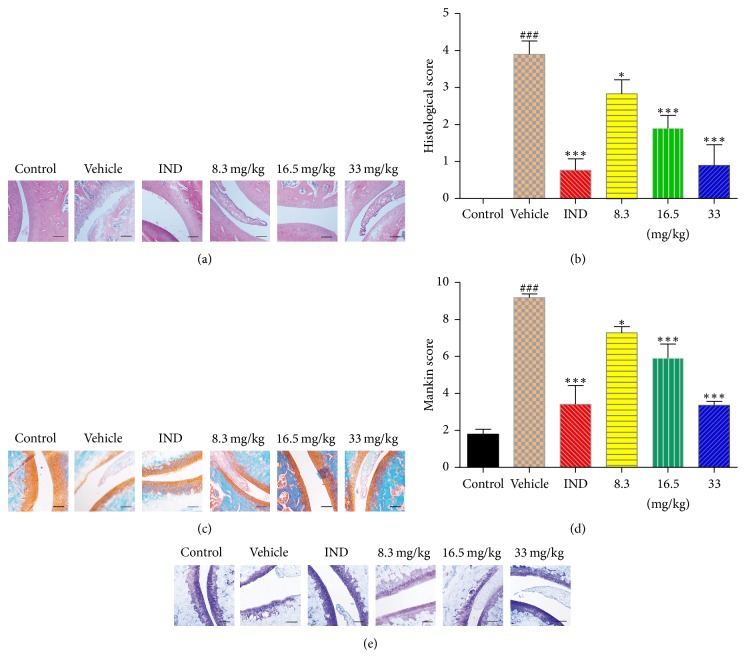
The antiadjuvant arthritis effect of toonaciliatin K on SD rats. Toonaciliatin K was injected using tail vein at the dosage of 8.3, 16.5, or 33 mg/kg each three days in total of 27 days of administration. The same volume 5% DMSO/saline was injected into vehicle. The IND (20 mg/kg) was administered orally once a day as positive. There was no intervention to control group. After 27 days, rats were sacrificed. (a) HE staining of cartilage; scale bar = 300 *μ*m. (b) The arthritis histological scores regarding HE staining. (c) Safranin O-fast green staining of cartilage; scale bar = 300 *μ*m. (d) The Mankin scores regarding safranin O-fast green staining. (e) The toluidine blue staining of paw sections; scale bar = 300 *μ*m. Statistical differences in different concentrations were considered significant at the levels of ^*∗*^*P* < 0.05 or ^*∗∗∗*^*P* < 0.001. The statistical differences among vehicle group and control group were considered significant at the levels of ^###^*P* < 0.001.

HE and safranin O-fast green staining demonstrated the impact of toonaciliatin K on histological changes, for example, synovial proliferation, inflammatory infiltrates, angiogenesis, edema, pannus formation, granuloma, focal loss of cartilage, bone erosion, and presence of extra-articular inflammation. HE staining results indicated that toonaciliatin K injection possessed a decreased trend in the bone histological score (Figures 4(a) and 4(b)). Meanwhile, safranin O-fast green staining results demonstrated that toonaciliatin K attenuated the cartilage damage in the test group (Figure 4(c)). Mankin's score consists of 4 aspects based on safranin O-fast green staining results: structure changes, cellular changes, safranin staining, and tidemark. Full thickness cartilage was found in control, while FCA induced apparently defects in the model. Toonaciliatin K injection decreased total Mankin's scores in a dose-dependent manner (Figure 4(d)). Moreover, representative histochemistry images showed intense toluidine blue staining in control group. However, toluidine blue staining about cartilage was remarkably decreased in the vehicle group compared with control group, which hinted the decline of proteoglycans and GAG content in the adjuvant arthritis. Intact cartilage surface was relieved by toonaciliatin K in a dose-dependent manner (Figure 4(e)).

## 4. Discussion

RA is well-known chronic inflammatory disorder which can damage human joint such as hand and feet with the characteristics of bone destruction, synovium inflammation, and cartilage damage. The persistent and chronic inflammation in the synovial membrane plays critical roles in the pathological basis of RA. Accumulating reports suggested that limonoids exhibited widely anti-inflammatory effect in LPS-induced RAW264.7 cells [37], carrageenan-induced acute paw edema [38], and D-galactosamine-induced liver injury [16]. However, the reports of antirheumatoid arthritis effect limonoids were still few until now. This study was to explore the anti-inflammatory effect of a type of limonoids, toonaciliatin K, in LPS-induced RAW 264.7 cell. The underlying mechanism was investigated. Furthermore, the anti-inflammatory effect of toonaciliatin K was evaluated on the carrageenan-induced edema. Finally, the antiarthritis effect of toonaciliatin K is investigated.

LPS-stimulated RAW 264.7 cell is commonly employed as anti-inflammatory screening model [39]. After LPS stimulation, macrophages produced excess response mediators such as prostaglandins, TNF-*α*, and IL-6. These inflammatory mediators can elicit the growth and dissemination of invading pathogens. Moreover, excess inflammatory mediators can result in microcirculatory dysfunction, septic shock, and inflammation. In this study, toonaciliatin K exhibited anti-inflammatory effect in LPS-induced RAW264.7 cells by inhibition of inflammatory mediators.

Moreover, carrageenan-induced edema is a representative acute inflammatory animal model used to investigate the anti-inflammatory effect of candidate drug. The acute inflammation in body can be divided into 2 phases. The primary acute phase (0–2 h) is a production process of histamine, kinins, and serotonin. The secondary subacute phase is a release process of bradykinin, leukotrienes, and prostaglandins [40]. Herein, there was only one statistical difference in the high dose group compared with vehicle group in the paw edema from 0 h to 2 h after carrageenan injection. This result indicated that toonaciliatin K inhibited the paw edema weakly in the first 2 h. However, 3 h later, toonaciliatin K with 3 doses inhibited the paw edema significantly. These results indicated that the toonaciliatin K mainly inhibited the secondary subacute phase in the carrageenan-induced edema.

The adjuvant arthritis in rat is a commonly used model with similar histology and immunology characteristic of human [41]. FCA injection elicited the excess proinflammatory cytokines production, paw swelling, and joint function loss in the adjuvant arthritis. The internal structures of joint such as cartilage, bone, and synovium are gradually damaged in the chronic adjuvant arthritis. Besides the effect of toonaciliatin K on macrophages, the effect of toonaciliatin K for cartilage was investigated using an adjuvant arthritis model elicited by FCA injection. Toonaciliatin K alleviated the histological changes of cartilage, which demonstrated the satisfied antiadjuvant arthritis effects of toonaciliatin K.

Many cytokines derived from macrophages, for example, TNF-*α*, IL-6, and IL-1*β*. The cytokines play key roles in the pathologic process of RA. Excess proinflammatory cytokines can provide a positive bride between fibroblast- and macrophage-like synoviocytes in RA [42]. Inhibition of inflammatory cytokines is the most common molecular target in RA's treatment. In this study, FCA injection resulted in the excess production of proinflammatory cytokines in adjuvant arthritis. However, the toonaciliatin K administration reduced the proinflammatory cytokines levels.

The iNOS is a critical enzyme in the regulation of inflammatory processes. The NO is a vasodilator which can block the adhesion of neutrophils to the vascular endothelium [43]. Usually, NO is maintained in a relatively low level. However, excess NO is produced after the activation of iNOS in cells and infiltrating leucocytes when inflammation is elicited in body tissue. The COX-2 is another important enzyme in the mediation of inflammatory processes [44]. Excess COX-2 leads to the production of PGE2 which can mediate the pain. In this study, toonaciliatin K suppressed the iNOS and COX-2 expression.

Many signaling pathways could regulate anti-inflammatory cellular response such as NF-*κ*B, AKT, and MAPK [45]. Accumulating report revealed that MAPK routes play important roles in production of COX-2 and iNOS mRNA and protein expression [35, 46]. After phosphorylation of MAPK pathways triggered by LPS, numerous cellular mediators were emerged for consequent inflammation response [47]. Meanwhile, the NF-*κ*B signaling pathway is also involved in the mediating of iNOS and COX-2 expression. Blocking of NF-*κ*B signaling pathway inhibited the syntheses of iNOS and COX-2. In addition, multiple proteins in the NF-*κ*B signaling pathway take part in the host defense response against harmful pathogen [48]. The NF-*κ*B signaling pathway mediates the proinflammatory cytokines production such as TNF-*α*, IL-6, and IL-1*β*. Downregulation of NF-*κ*B and MAPK could inhibit the inflammatory activity so they become the molecular target of numerous antiarthritis drugs. The AKT signaling pathway is commonly associated with some inflammatory disease via combination or individual regulation with MAPK or NF-*κ*B signaling pathway [49, 50]. However, the AKT signaling pathway is activated in LPS-stimulated RAW264.7 cells but not affected with toonaciliatin K. In this study, the NF-*κ*B and MAPK signaling pathways contributed combined regulation to the anti-inflammatory effect of toonaciliatin K.

Taken together, our assay manifested anti-inflammatory activity of toonaciliatin K in LPS-induced RAW264.7 cells in vitro and antiadjuvant arthritis effect in vivo. Toonaciliatin K's actions on RAW264.7 cells were regulated with MAPK and NF-*κ*B signaling pathways. This study supplied the pharmacology basis of toonaciliatin K as a promising agent for RA therapy.

## Figures and Tables

**Table 1:** Real-time PCR primer sequences used in mRNA levels assay in research in vitro.

Gene	Primer sequence
iNOS	Forward: 5′-GCA GAA TGT GAC CAT CAT GG-3′
	Reverse: 5′-ACA ACC TTG GTG TTG AAG GC-3′

COX-2	Forward: 5′-CAG GAA ATC CTT GCT GTT CC-3′
	Reverse: 5′-TGG GCA AAG AAT AAC ATC-3′

TNF-*α*	Forward: 5′-TAC TGA ACT TCG GGG TGA TTG GTC C-3′
	Reverse: 5′-CAG CCT TGT CCC TTG AAG AGA ACC-3′

IL-6	Forward: 5′-CCG GAG AGG AGA CTT CAC AG-3′
	Reverse: 5′-GGA AAT TGG GGT AGG AAG GA-3′

IL-1*β*	Forward: 5′-CCC TGC AGC TGG AGA GTG TGG-3′
	Reverse: 5′-TGT GCT CTG CTT GAG AGG TGCT-3′

GAPDH	Forward: 5′-ACC ACA GTC CAT GCC ATC AC-3′
	Reverse: 5′-CAC CAC CCT GTT GCT GTA GCC-3′

## Abbreviations

RA: Rheumatoid arthritis
LPS: Lipopolysaccharide
DMEM: Dulbecco's modified eagle's medium
FBS: Fetal bovine serum
NO: Nitric oxide
TNF-*α*: Tumor necrosis factor alpha
IL-6: Interleukin-6
IL-1*β*: Interleukin-1*β*
MAPK: Mitogen-activated protein kinase
NF-*κ*B: Nuclear factor-kappa B
iNOS: Inducible nitric oxide synthase
COX-2: Cyclooxygenase-2
HE: Hematoxylin-eosin
DEX: Dexamethasone
ELISA: Enzyme-linked immune-sorbent assay
IND: Indomethacin
FCA: Freund's complete adjuvant
ERK: Extracellular regulated protein kinases
JNK: c-Jun N-terminal kinase
PI3K: Phosphoinositide 3-kinase.
